# Duality of the Brain

**Published:** 1878-05-20

**Authors:** J. Barr Smith


### DUALITY OF THE BRAIN.

**Read before the Meigs Co. Medical Society.**

By J. Barr Smith, M. D.

*Mr. President and Gentlemen:*—As you are all familiar with the anatomy of the brain, I will occupy no more of your time with the anatomical construction than will be necessary to illustrate my position.

1st. The brain is a duality.

2nd. Each one of the pair is separate, distinct and complete in itself. Each one of the pair performs all its functions independent of its fellow. The functions of the brain are: the perception of an impression, volition, memory, and the power of originating motion. Therefore, each one of the pair has a faculty of the perception of an impression, volition, memory, and the power of originating motion.

Reasoning from analogy: Physiologists for the sake of convenience usually divide the body into a number of systems, each system consisting of a number of organs subservient to the same purpose in the economy. These organs are always found in pairs, except the outlets and reservoirs, the functions of which are passive, as they perform no active duty; for instance, we have two arms, each one of the pair complete in itself, capable of performing the same offices alone and unassisted by its fellow. Each arm has the same number of bones, muscles, etc., arranged in the same manner; so also of the lower extremities; so also of the organs of our senses, hearing, seeing, etc. The nerves are given off in pairs; the bronches. Now, when all the organs of the body of which we have positive knowledge are found in pairs, is it not reasonable to infer that the brain also consists of a pair separate, distinct and independent as the arms.

Reasoning from cause to effect and vice versa from effect back to cause: One of the pair of brains can and does recall impressions its fellow never had any perception of. We can and do call into activity one of the pair whilst its fellow is dormant. Each one of the pair may and does receive distinct impressions at the same time. A writing master can trace with a pen on paper a letter of the alphabet that will extend exactly to a certain line above and to a given line below; he can then make an exact copy of it, or a dozen if he wishes, so much alike you cannot detect any difference. To accomplish this he must judge of the exact distance to move his hand, the exact directions to move his hand, and the exact position to hold the pen. The power of originating motion is a faculty of the brain, not of the hand nor arm. His hand, therefore, moves as directed by the brain—the result of education. Let a man who has never learned to write undertake in like manner to make a dozen copies of the same letter. His arms may be as strong, his nerves as steady, his brain as active as the writing master. He may have all the physical qualifications, yet, he cannot make two letters exactly alike; it must be learned. Now let the same writing master take the pen in his left hand and undertake to make a copy of the letters he has traced with his right hand, and he can no more do it than the man who has never learned to write. Why? The nerves that supply the right arm are given off from one of the pair of the brains, the nerves distributed to the left arm from the other one of the pair. One of the pair of brains has been educated to move the hand the required distance and direction, whereas the other one of the pair has not been so educated.

If a man has only one brain, one memory, one source of originating motion, the distance and direction learned, the brain could and would direct the motion of either hand indiscriminately to move the required distance and direction. The writing master has educated but one of the pair of brains—the one correlated to the right arm, consequently to write with the left hand he must also educate the one of the pair correlated with left arm's hand, and this is true of all the organs of the body. Dancing masters frequently find their pupils learn to execute all the movements they require with one foot readily, and almost impossible to learn and execute the proper movements with the other foot of the pair. Now if the brain is a unity; if the pupil has but one memory, one source of originating motion, one volition, the time distance and direction once learned, the feet moving only as directed by the brain would of course be equal, the same time, distance and direction being required.

Again, we have a pair of organs communicating sound to the brain—one communicating directly with the right the other with the left brain. The time as well as the volume or force with which it reaches the brain must vary according to the direction from which the sounds proceeds and the position of the organs of hearing. If the brain is a unity this would destroy the harmony. Either one of the pair of brains has prehension of the sound and the other has not, or each pair receives the sound independent of its fellow. So also of sight, the rays of light from one object impinge on both eyes, two images are conveyed to the brain. If the brain is a unity we would of course see every object double.

Again, each one of the pair of brains can be active at the same time. A school teacher can add up a column of figures correctly and listen at the same time to a scholar recite a lesson. A man can write a letter on one subject, and at the same time listen to a conversation on a different subject. A man could as easily move one foot or one hand in different directions at the same time as one brain could act in different directions at the same time—reasoning from analogy.

The power of originating motion is a faculty of the brain, when the brain is injured so that the motion of a part is interfered with. If but one of the pair of brains is injured the paralysis is always on the side correlated to the brain injured. If the brain is a unity it would be impossible to paralize one arm without at the same time paralyzing both. Unilateral sweating from one of the pair being diseased could not result if the brain is not a duality.

Reasoning from the anatomical construction of the brain: The normal brains of two individuals differ in size, number and arrangement of convolutions, depth of anfractuosities as well as intellectual power and disposition. So also do the pair of brains differ in the same individual; the size, convolutions, etc., of the right differs from the left. By keeping in mind that the brain is a duality, and the pair differ in their formation in the same individual, we can easily understand the many apparent contradictions we find in the manner, disposition, etc., of the same individual.

RECAPITULATION.

1st. The brain is a duality because all the organs of the body of which we have positive knowledge are found in pairs, except the outlets and reservoirs.

2nd. One of the pair of brains may have the perception of an impression when its fellow has not.

3rd. One of the pair may be educated and the other not.

4th. Each one of the pair may have the perception of different impressions at the same time.

5th. When only one of the pair is injured so as to destroy the power of originating motion, the paralysis is only on one side of the body.

6th. The shape, size, convolutions, anfractuosities, etc., of one side differs from its fellow.

7th. We can in no other way account for the contradictions in men's characters, and the peculiarities of many diseases.—*Lancet and Observer.*

